# Alpha Traveling Waves during Working Memory: Disentangling Bottom-Up Gating and Top-Down Gain Control

**DOI:** 10.1523/JNEUROSCI.0532-24.2024

**Published:** 2024-11-06

**Authors:** Yifan Zeng, Paul Sauseng, Andrea Alamia

**Affiliations:** ^1^Department of Psychology, Universität Zürich, Zürich 8050, Switzerland; ^2^Cerco, CNRS Université de Toulouse, Toulouse 31059, France; ^3^ANITI,Université de Toulouse, Toulouse 31062, France

**Keywords:** alpha oscillations, electroencephalography, traveling waves, working memory

## Abstract

While previous works established the inhibitory role of alpha oscillations during working memory maintenance, it remains an open question whether such an inhibitory control is a top-down process. Here, we attempted to disentangle this issue by considering the spatiotemporal component of waves in the alpha band, i.e., alpha traveling waves. We reanalyzed two pre-existing and open-access EEG datasets (*N* = 180, 90 males, 80 females, 10 unknown) where participants performed lateralized, visual delayed match-to-sample working memory tasks. In the first dataset, the distractor load was manipulated (2, 4, or 6), whereas in the second dataset, the memory span varied between 1, 3, and 6 items. We focused on the propagation of alpha waves on the anterior-posterior axis during the retention period. Our results reveal an increase in alpha-band forward waves as the distractor load increased, but also an increase in forward waves and a decrease in backward waves as the memory set size increased. Our results also showed a lateralization effect: alpha forward waves exhibited a more pronounced increase in the hemisphere contralateral to the distractors, whereas the reduction in backward waves was stronger in the hemisphere contralateral to the targets. In short, the forward waves were regulated by distractors, whereas targets inversely modulated backward waves. Such a dissociation of goal-related and goal-irrelevant physiological signals suggests the coexistence of bottom-up and top-down inhibitory processes: alpha forward waves might convey a gating effect driven by distractor load, while backward waves may represent direct top-down gain control of downstream visual areas.

## Significance Statement

When exploring the functional role of alpha-band neural oscillations during working memory, mostly amplitude modulations have been considered so far, with relatively limited exploration of spatiotemporal dynamics of this rather global brain oscillatory signature. The present study seeks to address this gap by examining the directionality of alpha wave propagation during working memory retention. Our findings offer novel insights into the well-established inhibitory role of alpha waves, demonstrating that this function is manifested differently according to their propagation directions: forward waves seem to facilitate bottom-up gating, while backward waves might mediate top-down gain control.

## Introduction

Alpha waves (8–12 Hz) can easily be observed in the human electroencephalogram (EEG), and they are particularly well-studied brain signals. Studies connect alpha waves to working memory, especially during the retention phase (Sauseng et al., 2009; Riddle et al., 2020; Chen et al., 2023). Alpha power increases (1) with higher working memory load (Jensen et al., 2002), (2) when distractors are anticipated (Bonnefond and Jensen, 2012; Payne et al., 2013), and (3) when it is modulated by perceptual load of stimuli processed in working memory (Noonan et al., 2018; Gutteling et al., 2022; Jensen, 2024). These findings suggest an important role of alpha waves in shielding working memory from competing yet irrelevant stimuli. However, it remains unclear whether such inhibitory shielding process involves top-down rather than bottom-up control (Jensen, 2024). Potentially, this question could be resolved by considering alpha oscillations as traveling waves, i.e., taking spatiotemporal dynamics into account. This could inform us on the potential modulation of excitability across cortical areas in a coordinated way.

When considering the propagation of alpha waves throughout the cortex, it is important to acknowledge alternative explanations of coherent spatial phase gradients, such as overlapping local sources or rotating dipoles (Hindriks et al., 2014; Orczyk and Kajikawa, 2022; Zhigalov and Jensen, 2023). However, as shown by Freeman et al. (2000, 2003) and Alexander et al. (2016), scalp EEG can truly measure the actual cortical spatiotemporal dynamics. Particularly, as proposed by Zhigalov and Jensen (2023), local neural dynamics cannot account for the systematic anterior-posterior direction of global traveling waves. Subtle task-related changes in local sources would radically alter the direction of observed global waves if they arose in an artifactual manner due to projections of local activity onto the scalp.

Previous studies corroborated the hypothesis that alpha-band traveling waves along the anterior-posterior axis correlate with distinct cognitive functions. Event-related potentials, such as P2 and N2, have been shown to correspond to single-trial traveling alpha waves along the anterior-posterior axis (Alexander et al., 2006, 2009). Recently, it was shown that alpha backward waves (anterior to posterior) were lateralized to the hemisphere contralateral to the unattended visual field, suggesting a top-down inhibitory mechanism, while forward waves (posterior to anterior) increased in the hemisphere contralateral to the attended location, indicating their relation to visual processing (Alamia et al., 2023). Zhang et al. (2018) found alpha forward waves during a working memory task correlating with task performance. Whereas Halgren et al. (2019) observed alpha backward waves in intracortical recordings of epilepsy patients during quiet wakefulness, scalp EEG recordings in healthy participants (Pang et al., 2020) indicate both forward and backward propagation patterns: visual stimulation elicited forward waves, while lack of stimulation elicited backward waves. These studies pave the way for a potential dissociation between distinct candidate mechanisms underlying alpha inhibition in working memory. The systematic observations of traveling waves along the anterior-posterior axis point to their veridicality, as this effect has no easy explanation in terms of localized oscillatory sources or waves.

Although alpha-band traveling waves have been studied in visual perception and attention, their relation to working memory processes remains less explored. Here, we reanalyzed two publicly available EEG datasets that tested visual working memory paradigms. The first dataset manipulated the distractor load (Feldmann-Wüstefeld and Vogel, 2019), and the second dataset varied the set size of memory items (Adam et al., 2018). Using an adapted method from previous studies (Alamia and VanRullen, 2019), we computed the normalized power of alpha waves propagating in forward and backward directions. Our results revealed a dissociation between the distractor load effects, predominantly observed in forward waves, and the memory load effects, more pronounced in backward waves. These results indicate two distinct alpha-band phenomena related to inhibitory processes: forward waves gate the visual stream, while backward waves contribute to general top-down gain control.

## Materials and Methods

### Experiment design

#### Data download

Two publicly available EEG datasets were used. The first dataset has been provided by Feldmann-Wüstefeld and Vogel (2019) and downloaded from OSF (https://osf.io/a65xz/). The second dataset comes from Adam et al. (2018) and is also available on OSF (https://osf.io/8xuk3/).

#### Participants

##### Dataset 1

The study by Feldmann-Wüstefeld and Vogel (2019) consisted of three separate experiments, including 31 (13 males, 13 females, *M *= 21.2 years, SD = 2.6; the demographic information of five participants is missing in the dataset), 37 (16 males, 16 females, *M *= 25.0 years, SD = 3.7; the demographic information of five participants is missing), and 36 participants (18 male, 18 females, *M* = 22.9 years, SD = 3.2), respectively, resulting in a total of 104 volunteers. Data were recorded with the written understanding and consent of each participant.

##### Dataset 2

The dataset by Adam et al. (2018) consisted of two experiments with 31 (19 males, 12 females, *M* = 22.0 years, SD = 3.7) and 45 participants (24 males, 21 females, *M* = 21.7 years, SD = 4.2), respectively. In total, 76 participants were recorded in the original experiments and gave informed consent.

#### Experimental procedure

##### Dataset 1

**Figure 1.**
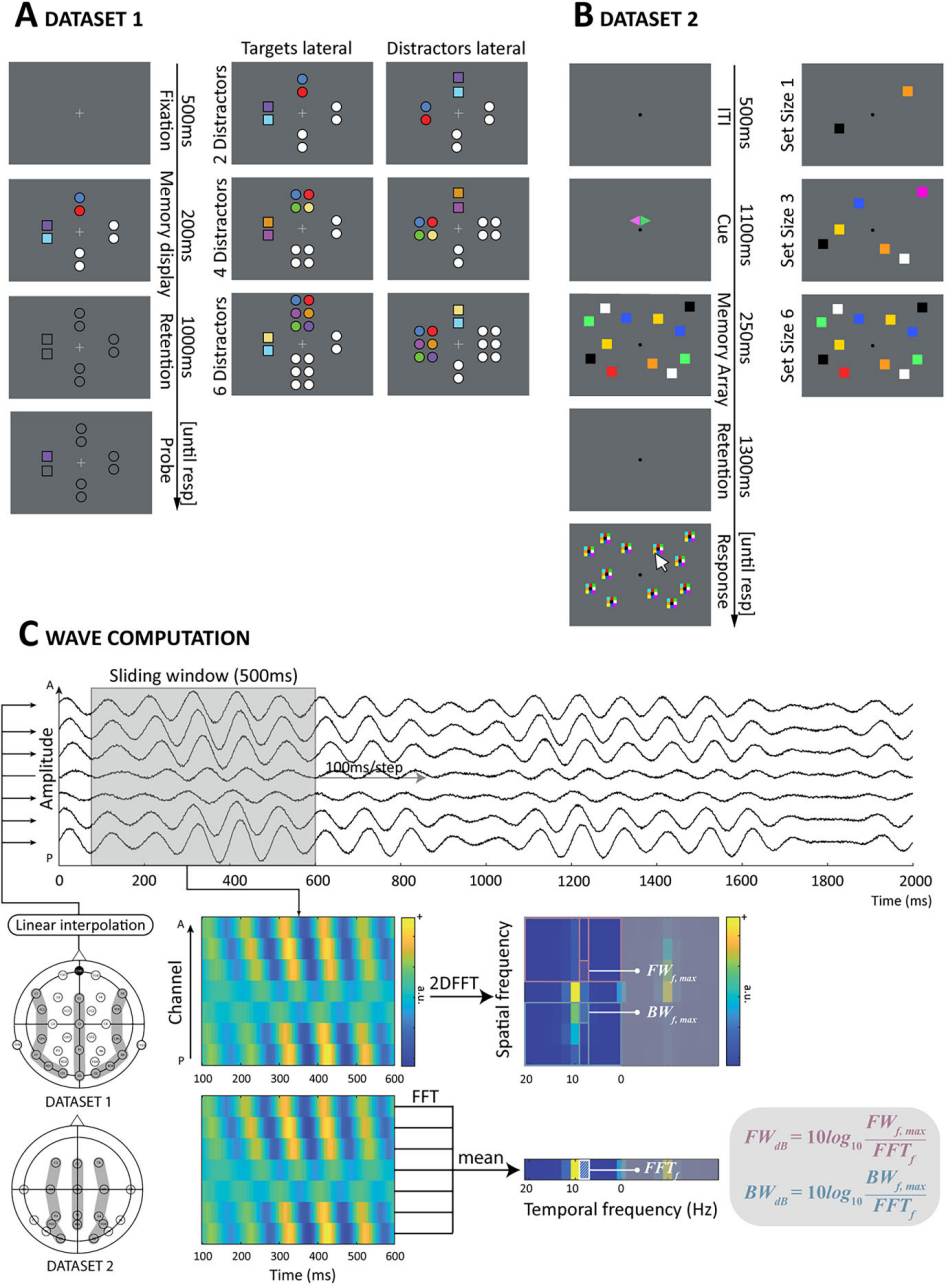
The experimental paradigms and wave computation method. ***A***, The change detection task of Dataset 1 (Feldmann-Wüstefeld and Vogel, 2019). Participants were required to keep in mind the colors of targets (squares) while ignoring the other distracting stimuli (circles). After a short retention interval, participants indicated whether the probe's color matched with the target presented at the same location. ***B***, The lateralized whole-report task of Dataset 2 (Adam et al., 2018). Participants were instructed to pay attention to and remember the stimuli in the hemifield cued by the green triangle while ignoring the rest. Then, they reported all the colors of the cued stimuli by clicking the color within the matrix corresponding to the items previously presented. ***C***, Wave quantification method. We selected three electrode axes; midline, left, and right hemisphere. The channels within each axis were linearly interpolated into seven channels and sorted according to their spatial location (from posterior to anterior). We employed a 500 ms sliding window at the step of 100 ms, and the resulting segments were fed into two-dimensional fast Fourier transform. In the power spectrum, the power of forward waves falls into the top-left (or down-right) quarter, whereas that of the backward waves falls into the down-left (or top-right) quarter. For a given temporal frequency and traveling direction, we defined the power as the maximum value in the corresponding column of the respective quarter, normalized by the averaged fast Fourier transform power of the same temporal frequency. A, anterior; P, posterior; 2DFFT, two-dimensional fast Fourier transform; FFT, fast Fourier transform; FW, forward waves; BW, backward waves.

In each experiment, a similar change detection task was used. The task included a memory display consisting of three types of stimuli: colored squares (targets), colored circles (salient distractors), and gray circles (placeholders). Clusters of targets, salient distractors, and two groups of placeholders were presented in one of four different positions (Fig. 1*A*). In half of the trials, the target cluster was presented in the left or right position, and the salient distractor cluster was presented at the top or bottom (i.e., “targets lateral” condition). In the other half of the trials, the positions of targets and distractors were reversed (i.e., “distractors lateral” condition). The number of targets was kept constant while varying numbers of colored distractors were presented in separate trials: distractor load 2 and 4 in Experiment 1; distractor load 2, 4, and 6 for Experiment 2; and heterogeneous (four distractors in four different colors) and homogeneous distractors (four distractors in two colors, not used in our study and, thus, not displayed in Fig. 1*A*) for Experiment 3.

Each trial started with a central fixation cross of 500 ms. Then, the memory display was presented for 200 ms, followed by a retention interval of 1,000 ms. A probe followed and was displayed until participants responded. The participant's task was remembering each target's color and location while ignoring the distractors. After the probe was presented, they had to indicate whether the color and location of the probe matched the previously presented targets. The trial ended with the participants responding (button press) and a 1,000 ms blank screen as the intertrial interval. See Feldmann-Wüstefeld and Vogel (2019) for more detail.

##### Dataset 2

The two experiments from Dataset 2 were lateralized whole-report tasks in which participants reported the color of a subset of previously presented targets. The memory display contained two lateralized stimulus clusters, with equal number of squares on each half of the screen. For each cluster, the squares were randomly distributed within one hemifield, and each square color was chosen without replacement from a pool of nine colors (red, green, blue, yellow, magenta, cyan, orange, white, and black). Repetition of the colors was allowed between hemifields. Before the memory display, a diamond-shaped cue was presented above the central fixation point. The cue was composed of a green and a pink triangle, and participants were instructed to pay attention to the visual hemifield cued by the green triangle. In Experiment 1, the number of items in each cluster varied between 1, 3, and 6. In Experiment 2, it was kept at a constant level of 6 (Fig. 1*B*).

Each trial began with a 500 ms fixation point. A small diamond-shaped cue of 1,100 ms was then presented above the fixation point. A brief memory display lasting 250 ms was presented, and participants were instructed to maintain fixation on the fixation point while encoding information presented in the visual hemifield indicated by the green half of the cue. The memory display was followed by a retention period of 1,300 ms. Participants were required to memorize the colors of each item on the cued side during the retention period, and they reported them by clicking the corresponding color within a 3 × 3 matrix (containing all nine possible colors) presented afterward at each target location. In each trial, the test period lasted until participants had responded to all target items. Finally, participants initiated a new trial with an additional mouse click.

#### EEG acquisition and preprocessing

##### Dataset 1

EEG was recorded using a 32-channel Brain Products actiCap (electrodes positioned according to the International 10/10 System: FP1/2, F7/8, F3/4, Fz, FC5/6, FC1/2, C3/4, Cz, TP9/10, CP5/6, CP1/2, P7/8, P3/4, PO7/8, PO3/4, Pz, O1/2, Oz) with a sampling rate of 1,000 Hz. Horizontal and vertical EOGs were recorded. All electrodes were referenced to TP10 and rereferenced offline to the average of all electrodes. For each trial, the dataset provided segmented data from 800 ms before to 1,600 ms after the onset of the memory display. We did not conduct any further preprocessing going beyond the preprocessing already applied in the provided dataset.

##### Dataset 2

EEG during the whole-report task was recorded using a 20-channel electrodes cap (Electro-Cap International) from standard 10/20 sites: F3, Fz, F4, T3, C3, Cz, C4, T4, P3, Pz, P4, T5, O1, and O2. Additionally, five nonstandard sites were included as follows: OL (midway between T5 and O1), OR (midway between T6 and O2), PO3 (midway between P3 and OL), PO4 (midway between P4 and OR), and POz (midway between PO3 and PO4). Horizontal and vertical EOGs were recorded to measure horizontal eye movements as well as blinks. All sites were recorded with a right mastoid reference and rereferenced offline to the algebraic average of the left and right mastoids. The sampling frequency was 250 Hz. The dataset contained raw segmented data (from 1,400 ms before the onset of the memory display to 1,548 ms after the memory display onset). The traveling wave analysis was performed without further preprocessing.

For both datasets, refer to the original publications by Adam et al. (2018) and Feldmann-Wüstefeld and Vogel (2019) for additional details regarding the presentation apparatus, presentation parameters, pretest (a change detection task to measure working memory capacity), as well as further information about participants and EEG recording setup.

### Statistical analysis

#### Traveling wave computation

Similar analyses as described by Alamia et al. (2020) were used. Data was analyzed using custom Matlab R2022b (The MathWorks) scripts and the bayesFactor extension package (Krekelberg, 2023). The data was further exported to JASP (JASP Team, 2024) for Bayes factor statistical analysis.

Figure 1*C* depicts the quantification method of traveling waves. For each dataset, we selected three electrode axes: one in the midline (Dataset 1: Fz, Cz, Pz, and Oz; Dataset 2: Fz, Cz, Fz, Poz), one over the left hemisphere (Dataset 1: F7, FC5, CP5, P7, PO7, O1; Dataset 2: F3, C3, P3, PO3, O1), and one over the right hemisphere (Dataset 1: F8, FC6, CP6, P8, PO8, O2; Dataset 2: F4, C4, P4, PO4, O2). All selected axes had alpha power beyond the 1/*f* aperiodic power function, confirming oscillatory alpha activity in the signal. We linearly interpolated the channel array into seven channels to achieve a consistent number of channels across different axes and facilitate the subsequent wave computation. For each trial, we stacked the data of interpolated channels, arranging them from the posterior to anterior regions. Next, a 500 ms sliding window moved across the data in steps of 100 ms to capture a two-dimensional segment (seven interpolated channels by 500 ms). In the resulting data segments, traveling waves would manifest as planar waves tilted at different angles (i.e., with a monotonic phase shift across channels).

Traveling waves were quantified by the two-dimensional fast Fourier transform (2DFFT), which was applied to the data segments considered as images. In the resulting power spectrum of the 2DFFT, the *x*-axis denotes the zero-centered temporal frequencies, while the *y*-axis represents the spatial frequencies. Keeping in mind that one of the 2DFFT's properties is to have conjugation symmetry, we considered the power of forward waves reflected into the top-left (or down-right) quarter, whereas that of the backward waves falls into the down-left (or top-right) quarter. The horizontal midline quantifies the power of standing waves. For a given temporal frequency and traveling direction, we defined the power as the maximum value in the corresponding column of the respective quarter. Yet, as with any other attempts to capture the phasic relationship of oscillations, the traveling wave measure at this stage is susceptible to the temporal oscillatory power, potentially confounding our results. We thus applied a normalization factor using the 1D-FFT power averaged across the seven channels composing each data segment (FW, forward waves; BW, backward waves):

$$FW_{\mathrm{dB}}=10\log_{10}\;\left(\frac{FW_{\;f,\max}}{\mathrm{FF}T_{f}}\right),$$

$$BW_{\mathrm{dB}}=10\log_{10}\;\left(\frac{BW_{\;f,\max}}{\mathrm{FF}T_{f}}\right).$$

The power in decibels enables a straightforward interpretation of the quantity of traveling waves by comparing them to zero, which serves as a baseline to account for power fluctuations. Subsequently, we computed the power of alpha-band traveling waves by averaging the power of frequencies ranging from 8 to 12 Hz. Furthermore, it is crucial to emphasize that our wave analysis concentrates on the sensor level. This choice is motivated by significant limitations associated with source projections, including the disruption of long-range connections and signal smearing caused by scalp interference (Nunez, 1974; Freeman and Barrie, 2000; Alexander et al., 2019).

#### Data analysis

To make the best use of the datasets, we pooled together the subexperiments within each dataset by their conditions. This led to a 2 (lateralized stimuli: “targets lateral,” “distractors lateral”) by 3 (distractor load: “load 2,” “load 4,” and “load 6”) design for Dataset 1, and three levels of set size (“set size 1,” “set size 3,” and “set size 6”) for Dataset 2.

For each dataset, we conducted three analyses: (1) a Bayesian repeated-measures ANOVA with distractor load (or set size for Dataset 2) as the independent variable for midline traveling waves; (2) Bayesian *t* tests comparing the power of alpha traveling waves between two lateral axes for each time point; (3) a Bayesian repeated-measures ANOVA with the factors of axes (i.e., wave power on the axis ipsilateral or contralateral to the items of interest) and distractor load (or set size for Dataset 2) as the independent variables. A description of additional supporting analyses can be located in the Results, Supplemental Analyses section.

Please note the inconsistency of study designs across subexperiments. For this reason, analyses (1) and (3) did not include all the available data (Table 1). For Dataset 1, only Experiment 2 was included, as it is the sole experiment with three levels of distractor load. For Dataset 2, only Experiment 1 was included, given it is the only experiment with three levels of set size.

**Table 1.** The analyses and utilization of datasets

Analysis No.	Dataset 1			Dataset 2	
	Experiment 1	Experiment 2	Experiment 3	Experiment 1	Experiment 2
1		√		√	
2	√	√	√	√	√
3		√		√	

Different analyses used data from different sets of experiments.

#### Code accessibility and open science

The code for traveling wave computation is available at https://osf.io/8tcw2/. This study has not been preregistered.

## Results

### Midline alpha traveling waves

As the first step, we examined the midline axes to investigate how manipulation of distractor load and set size modulate alpha traveling waves during the retention period irrespective of lateralization.

**Figure 2.**
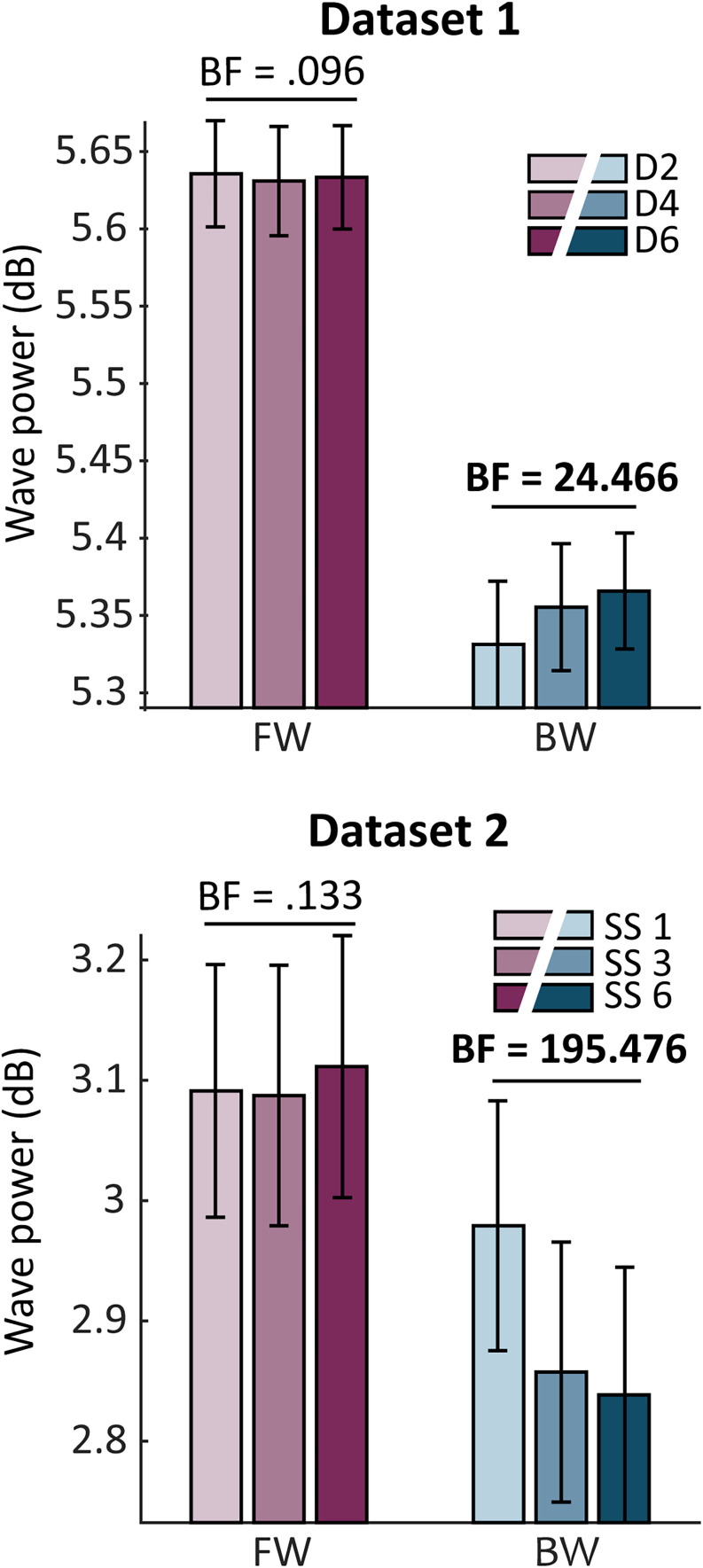
Midline alpha traveling waves during retention periods. Backward waves increased with distractor load in Dataset 1 and decreased with set size in Dataset 2. BF, the inclusion Bayes factor; D2/D4/D6, distractor load 2, 4, or 6; SS 1/3/6, set size 1, 3, or 6; FW, forward waves; BW, backward waves.

In Dataset 1, we found that the amount of midline alpha backward waves, but not forward waves, increased when the number of distractors increased (Fig. 2, top panel). Bayesian repeated-measures ANOVA revealed that the observed alpha power of backward waves was more likely based on the model with distractor load than the null model (BF_incl_ = 24.466). These results suggested that midline alpha backward waves increased when more distractors were presented. Post hoc comparisons indicated substantial evidence for differences between “load 2” and “load 4” (Δ*M *= −0.024; BF_10_ = 4.670; error = 4.477 × 10^−7^%) and “load 2” and “load 6” *(*Δ*M *= −0.035; BF_10 _= 13.415; error = 7.800 × 10^−8^%). Between “load 4” and “load 6,” we found some evidence in favor of the null hypothesis (Δ*M *= −0.011; BF_10 _= 0.382; error = 0.036%). In contrast, we found strong evidence against the effect of distractor load on midline alpha forward waves (BF_incl_ = 0.096).

In Dataset 2, we also found a modulation of backward waves but not of forward waves; differently than in Dataset 1, the midline alpha backward waves decreased with the number of items to be remembered (Fig. 2, bottom panel). Our analysis revealed stronger evidence for the effect of set size on midline alpha backward waves than the null hypothesis (BF_incl_ = 195.476); fewer backward waves were found when the set size increased. Post hoc comparisons showed significant differences between “set size 1” and “set size 3” (Δ*M *= 0.122; BF_10_ = 12.844; error = 4.627 × 10^−8^%), “set size 1” and “set size 6” (Δ*M *= 0.141; BF_10_ = 34.020; error = 1.673 × 10^−8^%), but weak evidence against the difference between “set size 3” and “set size 6” (Δ*M *= 0.019; BF_10_ = 0.284; error = 0.034%). These results indicate that the variation of set size also influenced midline alpha backward waves, yet in a different way: in the first dataset, backward waves increased with the number of distractors, whereas in the second dataset, backward waves decreased with set size. As for the midline alpha forward waves, our analysis revealed moderate evidence against a main effect of set size (BF_incl_ = 0.133).

### Lateral alpha traveling waves

Next, we focused our analysis on alpha-band traveling waves propagating in each hemisphere by comparing the power between ipsi- and contralateral axes with respect to the stimuli in Dataset 1.

**Figure 3.**
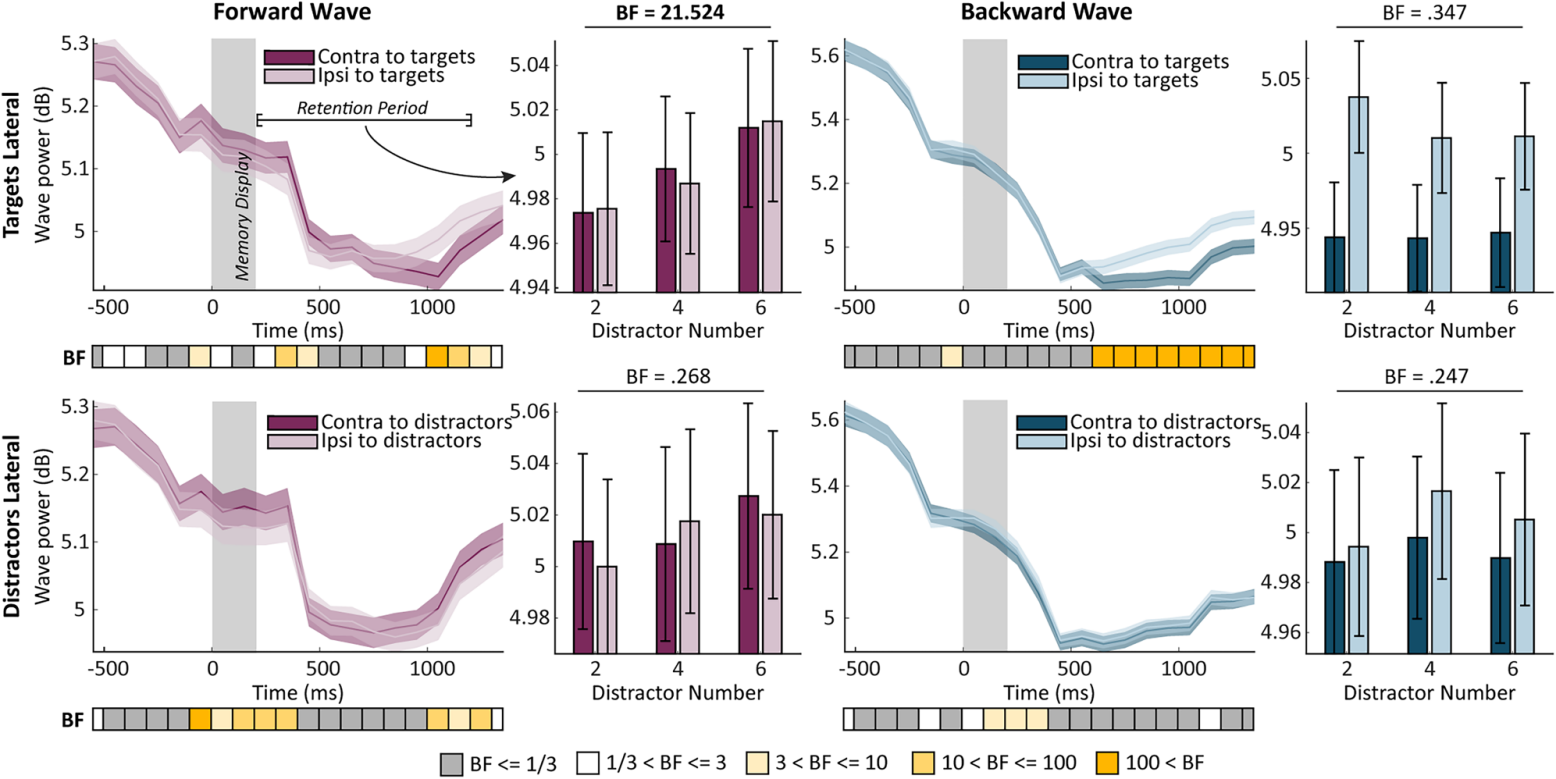
Bilateral alpha traveling waves in Dataset 1. Line plots: the power of bilateral alpha traveling waves across time. Below, the color bar indicates the Bayes factors of the power difference between two lateral axes for each time point. Bar plots: the wave power averaged over the retention period. The inclusion Bayes factors above the plots show the main effect of distractor load.

We first performed Bayesian *t* tests on contralateral versus ipsilateral wave power in each condition. The line graphs in Fig. 3 depict wave power at each time point. At the later stage of the retention period (1,050–1,250 ms), we found a higher alpha forward power in the axis contralateral to placeholders in the “target lateral” and to distractors in the “distractor lateral” condition—both are more distracting items compared with those in the opposite hemifield. In contrast, alpha backward waves were lateralized to the axis ipsilateral to the targets in the “targets lateral” condition, but we found no differences between the two axes in the “distractors lateral” condition.

**Figure 4.**
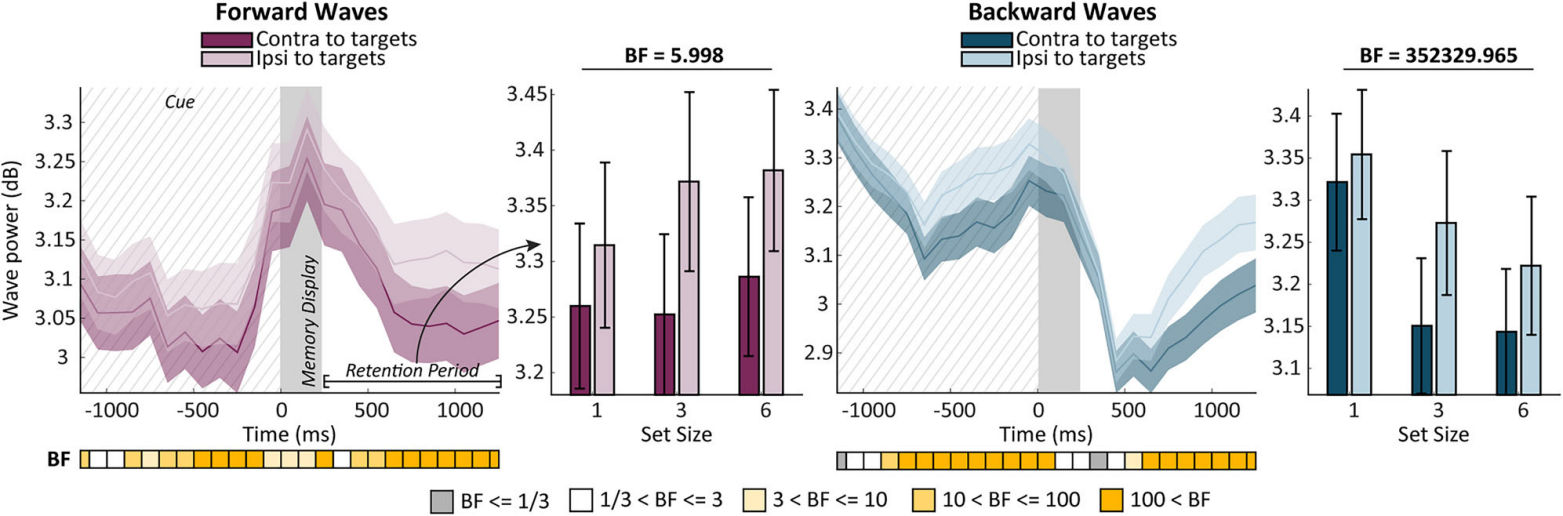
Bilateral alpha traveling waves in Dataset 2. The line plots and bar plots represent the bilateral wave power across time and averaged over the retention period, respectively. For more details, please refer to the legend of Figure 3.

In Dataset 2 (Fig. 4, line graphs), the comparison between the contra- and ipsilateral axes revealed similar lateralization patterns regardless of the traveling direction. Specifically, we observed an increase in forward and backward waves in the axis ipsilateral to the target side. Regarding alpha forward waves, this pattern persisted throughout almost the entire analysis window (all BF_10_ > 3), while for backward waves, the effect mainly took place during the cueing (−850–50 ms) and the retention period (550–1,250 ms).

#### Interaction of axes and effects of distractor load/set size

We then investigated whether the lateralization of traveling waves was modulated by distractor load (Dataset 1) and set size (Dataset 2). The analysis focused on the retention phase by averaging the wave power within these time windows (Dataset 1, 200–1,200 ms; Dataset 2, 250–1,550 ms).

In Dataset 1, we conducted four repeated-measures ANOVAs for each wave direction and lateralized stimuli (Fig. 3, bar plots). We considered axes, distractor load, and their interaction term axes * distractor load as predictors. Overall, the results revealed an increase in alpha forward waves in response to increased distractor load, yet only for “targets lateral” condition. Specifically, we found strong evidence for the main effect of distractor load (BF_incl_ = 21.524), whereas the main effect of axes and their interaction term remain in favor of the null hypothesis (all BF_incl_ < 0.173). As for the “distractors lateral” condition, we found no evidence in favor of the alternative models (all BF_incl_ < 0.268).

We did not observe a similar effect for alpha backward waves. In the “targets lateral” condition, the model only considering axes provided the best fit (the analysis of effect showed axes BF_incl_ = 2,285.956, whereas for distractor load BF_incl_ = 0.347 and for distractor load * axes BF_incl_ = 0.835). Regarding the “distractors lateral” condition, none of the effects provided conclusive evidence over the null hypothesis (axes, BF_incl_ = 0.726; distractor load, BF_incl_ = 0.247; axes * distractor load, BF_incl_ = 0.069). Overall, these results show that while the amount of alpha forward waves was influenced by distractor load in the “targets lateral” condition, this effect did not modulate backward waves.

In Dataset 2, we conducted two separate repeated-measures ANOVAs for each traveling direction, considering axes, set size, and the interaction axes * set size as predictors. The results are shown in the bar plots of Figure 4. In addition to axes (BF_incl_ = 53,885.652), which was already addressed via Bayes *t* tests in the section above, we found an increase of alpha forward waves with set size (BF_incl_ = 5.998). Our analysis also revealed an interaction between axes and set size (BF_incl_ = 16.855), suggesting that the increase of alpha forward waves was more prominent on the axes ipsilateral to the relevant side.

In contrast, our analysis showed that bilateral backward waves decreased while the set size increased. Analysis of effects showed decisive evidence for the main effects of set size (BF_incl_ = 352,329.965) and axes (BF_incl_ = 2,052.282). Moreover, the interaction between the two factors provided strong evidence (BF_incl_ = 24.979), indicating that the decrease of backward waves was more substantial contralateral to the target positions. All in all, these results showed that alpha forward waves increased when the overall number of items on the unattended side increased. In contrast, backward waves decreased with the number of items on the attended side.

#### Alpha-band power at ipsi- and contralateral axes

Other studies, such as Adam et al. (2018), investigated the role of (stationary) alpha amplitude in working memory; therefore, for the sake of completeness, in addition to the above-reported effects on traveling alpha waves, we also analyzed conventional alpha amplitude. In the first dataset, strong evidence was found for main effects of axes (BF_incl_ = 1.387 × 10^+11^) in “targets lateral” but not “distractors lateral” condition (BF_incl_ = 0.293). We also found strong evidence for the main effect of distractor load for both conditions (targets lateral: BF_incl_ = 2.126 × 10^+7^; distractors lateral: BF_incl_ = 2,603.310), suggesting decreased alpha amplitude with more distractors. The interaction effect for axes * distractor load for “targets lateral” also provided strong evidence (target lateral: BF_incl_ = 1,085.272; distractor lateral: BF_incl_ = 0.529). In the second dataset, we found strong effects of axes (BF_incl _= 5,652.873), set size (with lower alpha power for larger set sizes, BF_incl_ = 7,582.176) and for the interaction axes * set size (BF_incl_ = 32.071). In short, our analysis replicated the alpha effects commonly observed in lateralized visual displays. More importantly, the interaction effect in the second dataset suggested difference between the two axes increased with the set size. This is in line with the original analysis of Adam et al. (2018), where higher set sizes were associated with stronger lateralized alpha power suppression (contralateral–ipsilateral sites). Note that the main effects of set size and distractor load should be interpreted with extra caution, since an increase in distractor and target loads was confounded with also more visual stimulation in general. It is a trivial effect that stronger visual stimulation results in more alpha amplitude decrease over posterior recording sites.

### Supplemental analyses

#### Lack of relation between traveling waves and working memory capacity

We failed to find any relation between individual working memory capacity (i.e., *K* values) and the magnitude of traveling wave changes induced by different levels of distractor load/set size*.* The individual *K* values [*K *= set size × (hit − false alarm)] were computed from data of separate experiments (also available in the same open datasets) designated to measure individual working memory capacity. The change magnitude of traveling waves was defined by fitted slopes of traveling wave power against distractor load/set size. Bayesian linear regression showed evidence in favor of the null model regardless of datasets and traveling directions (all BF_10_ < 0.355).

#### Frequency specificity of traveling wave effects

The effects of traveling waves are alpha-specific. We conducted the identical analyses on the neighboring frequency bands [i.e., theta (4–7 Hz) and beta (13–30 Hz) band]. Most of the analyses favored the null model or failed to provide evidence for the alternative model regardless of traveling axis and direction (all BF_incl_ < 2.719). One exception lies in the “target lateral” condition of Dataset 1, where our analysis showed moderate evidence for distractor load effect for bilateral beta forward waves (BF_incl_ = 8.326) and strong evidence for axes difference for beta backward waves (BF_incl_ = 80.263). Note that these effects are similar to what is observed at alpha frequency, however, to a much smaller extent. We suggest this may be caused by alpha effects leaking into the neighboring (beta) frequency.

#### Robust effects across single trials

As statistical analyses are based on averages across multiple single trials per condition and per participant, one could argue that effects on traveling waves could potentially be driven by only a few extreme single trials. To rule this possibility out, we additionally conducted all the analyses as reported above based not on mean values across trials but based on median values across single trials. Using the trial median values yielded nearly identical results across all analyses.

## Discussion

**Figure 5.**
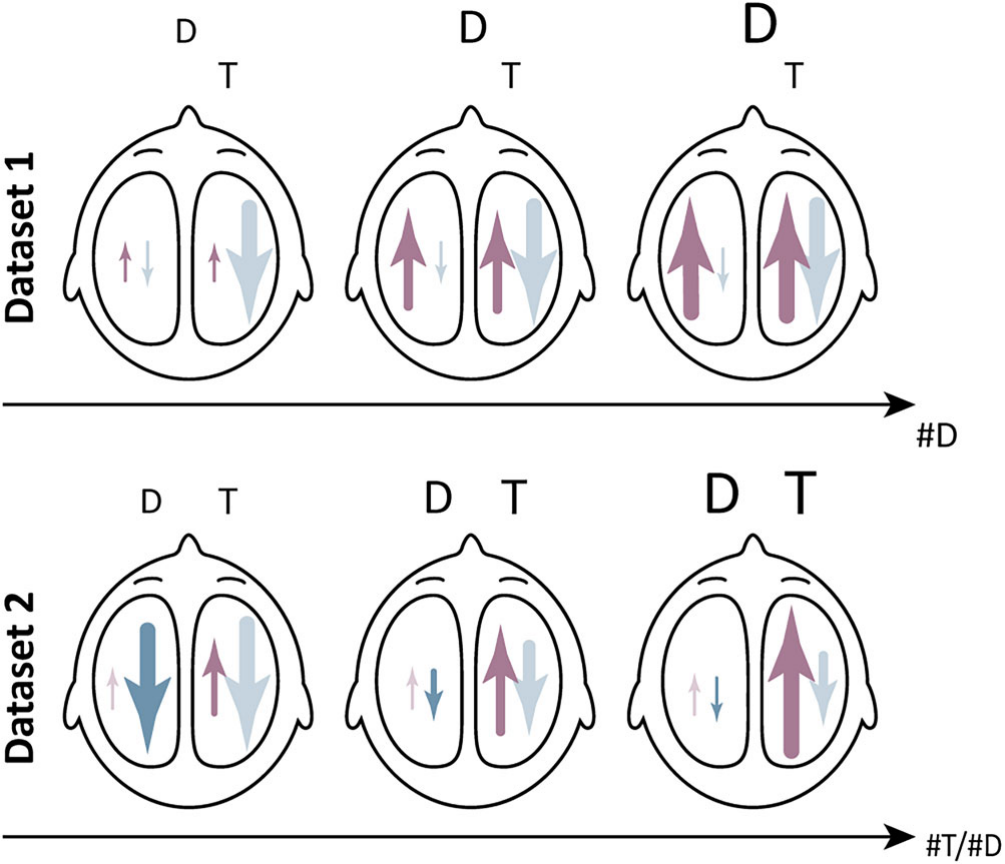
Schematic summary of the results. The size of the arrows indicates the power of traveling waves. We denote the effects of interests with less transparency. D, distractors; #D, distractor load; T, targets; #T, set size.

The present study aimed to investigate the functional roles played by forward and backward alpha-band traveling waves during working memory tasks. Specifically, we showed how different features manipulate working memory processes; namely, distractor loads (i.e., number of distractors, Dataset 1; Feldmann-Wüstefeld and Vogel, 2019) and set size (i.e., number of targets, Dataset 2; Adam et al., 2018) differentially modulate forward and backward waves. Figure 5 summarizes these results schematically. In Dataset 1, the alpha power of midline backward waves (not included in Fig. 5) and the power of bilateral forward waves increased with distractor load during the retention phase. In addition, we showed that alpha backward waves, but not forward waves, were lateralized to the axis ipsilateral to the encoded targets. In Dataset 2, we found that larger set size led to an increase in the propagation of bilateral forward waves and, conversely, a decrease of backward waves overall. These effects depend on the hemispheres where the waves propagate: the increase of forward waves was more prominent on the axis contralateral to the distractors, whereas the reduction of backward waves was more pronounced on the axis contralateral to the targets. The overall traveling wave amount, however, was higher on the axis contralateral to the distractors for both wave direction. In the following, we interpret our results in light of the differential roles of forward and backward alpha-band waves during visual working memory tasks.

### Alpha forward waves reflect gating during retention

Our findings in alpha forward waves go in line with the idea of alpha waves inhibiting irrelevant information by a gating mechanism (Jensen and Mazaheri, 2010). Specifically, in Dataset 1, we observed an increase in forward waves accompanying an increase in the central distractor load; assuming that stronger forward alpha waves represent gating of information flow from lower to higher visual areas, a higher distractor load would also require more of such gating. Accordingly, in Dataset 2, there was a similar effect observed for forward waves contralateral to distractors: an increase in the number of distractors went hand in hand with increasing forward waves, indicating more rigorous gating in the hemisphere contralateral to distractors. No such effect was observed, however, for the hemisphere contralateral to targets. As no targets should be filtered away, even at high target load, there was no increase of forward waves obtained in the contralateral hemisphere.

Such gating of sensory information (Jensen and Mazaheri, 2010) may not be under direct top-down control (Jensen, 2024), also indicated by the “forward” traveling direction in the present study. It has been suggested that the rhythmic fluctuation of alpha oscillations provides pulsed inhibitions to gate the flow of sensory input (Jensen and Mazaheri, 2010). As a result, information that fall into the excitatory phase (i.e., the duty cycle) is more likely to be processed than information during the phase of inhibition. Probably, the gating of information does not take place in early visual areas but instead somewhere slightly higher in the visual hierarchy (Peylo et al., 2021; Jensen, 2024). In contrast to that, here, we suggest that gating does not take place at only one level within the visual hierarchy; instead, we proposed that this gating mechanism is implemented by alpha forward waves, superimposing the low-level visual input and carrying out real-time screening along the information stream while it flows toward higher regions.

#### Distinct roles for stimuli-evoked and inhibitory alpha-band forward waves

However, it is worth noting that alpha-band forward waves may play distinct functional roles during different stages of the current working memory task (e.g., during memory display vs retention phase). A recent study suggested that alpha-band forward waves may appear during low-level visual stimulation, as the authors reported forward waves during visual stimulation but less so in the absence of sensory input (Pang et al., 2020). Similarly, during presentation of the memory display (Fig. 3), we observed more contralateral than ipsilateral forward waves in both “target lateral” and “distractor lateral” conditions, in line with the hypothesis that forward waves are increased by sensory stimulations, as both targets and salient distractors were perceptually more salient than the placeholders. In other words, the colorful stimuli provided more low-level visual information compared with the gray placeholder on the other side of the screen, which may lead to more alpha forward waves. However, more visual perception-related forward alpha waves might be difficult to dissociate from more memory-related forward alpha waves as we see them during the retention interval. In the current task they are observed during different task phases, though, and they respond differently to changes in target and distractor load. Possibly, more fine-grained spatial analysis (like suggested by Zhang et al., 2018) of traveling waves could prove successful for dissociating those functionally different waves in the future.

### Alpha backward waves reflect top-down gain control

In contrast to forward waves, alpha backward waves were not modulated by distractor load. Instead, backward waves were generally strong in the hemisphere ipsilateral to targets. This might indicate an inhibitory function of alpha backward waves in working memory, similar to effects obtained associated with spatial attention (Alamia et al., 2023). Moreover, this pattern is reminiscent of findings reported on stationary posterior alpha amplitude when spatial attention is shifted to one visual hemifield, resulting in increased alpha activity ipsilateral to the locus of attention (Worden et al., 2000; Sauseng et al., 2005; Thut et al., 2006; Medendorp et al., 2007; Foxe and Snyder, 2011).

In the hemisphere contralateral to targets, backward alpha waves are inversely related to target load. An increased number of targets led to a reduction of backward waves (Fig. 5). Considering alpha backward waves as an inhibitory top-down biasing signal here, a reduction of backward waves with increasing number of targets would lead to release of inhibition in more downstream, lower visual areas. Therefore, backward alpha waves in the here reported data might be a correlate of inhibitory gain control in the visual system.

### Linking past studies of alpha traveling waves

While a substantial body of literature has firmly established the inhibitory role of alpha waves during working memory performance (Klimesch et al., 2007; Sauseng et al., 2009; Jensen and Mazaheri, 2010; Khader et al., 2010; Bonnefond and Jensen, 2012; Wianda and Ross, 2019; Riddle et al., 2020; Gutteling et al., 2022; Jensen, 2024), the notion of alpha activity as traveling waves in the human neocortex has only recently been fully considered (Zhang et al., 2018; Soroka and Idiart, 2021). In line with our results, these recent studies have suggested alpha waves traveling at different propagation directions during the different stages of a memory task, not only within the prefrontal cortex (Bhattacharya et al., 2022) but also on a large scale (Mohan et al., 2022 provided evidence for the preferred traveling axis to be posterior to anterior). To our knowledge, the current study is the first one to investigate alpha traveling waves with respect to the load of relevant/distracting information during the retention period of working memory tasks. Applying the approach reported in the current study to retro-cuing visual working memory tasks could help investigate if alpha traveling waves may be involved in erasing already stored information, as suggested by Soroka and Idiart (2021). Overall, our results promote the current understanding of the link between large-scale alpha traveling waves and working memory.

### Conclusion

Our study may provide neural evidence for the coexistence of two parallel potential inhibitory mechanisms of alpha waves for the first time. These two mechanisms may be reflected by alpha waves, which propagate in opposite directions along the anterior-posterior axis: alpha backward waves may represent a direct top-down biasing signal for gain control, whereas forward waves might convey a bottom-up gating effect.

It is important to note that these results are unlikely to be caused by localized sources or waves (Hindriks et al., 2014; Orczyk and Kajikawa, 2022; Zhigalov and Jensen, 2023). Specifically, subtle changes in their position and orientation relative to sulci and gyri would induce a noise-like contribution to direction of propagation of alpha waves, if solely due to scalp projection of localized neural dynamics. In contrast, we find task-dependent systematic effects along the anterior-posterior axis, as has been found in many studies on traveling waves (Zhang et al., 2018; Halgren et al., 2019; Pang et al., 2020; Alamia et al., 2023), which favors an explanation in terms of global cortical phases gradients. One of the main conclusions of this work is the importance of considering not only the temporal but also the spatial dynamics when investigating neural oscillations. For example, considering only the power difference between the target- and distractor-induced effect might not capture the true complexity of how targets and distractors are processed in working memory. Similarly, we presume that considering oscillations as traveling waves propagating in different directions could shed light on some of the contradicting studies investigating the functional roles of alpha waves in working memory.
